# A health care system in transformation: making the case for chiropractic

**DOI:** 10.1186/2045-709X-20-37

**Published:** 2012-12-06

**Authors:** Richard Brown

**Affiliations:** 1 The Lansdown Clinic, 1, High Street, Stroud, Gloucestershire, GL5 1AU, United Kingdom

**Keywords:** Chiropractic, Healthcare transformation, Healthcare reform

## Abstract

There are a number of factors that have conspired to create a crisis in healthcare. In part, the successes of medical science and technologies have been to blame, for they have led to survival where lives would previously have been cut short. An informed public, aware of these technological advances, is demanding access to the best that healthcare has to offer. At the same time the burden of chronic disease in an increasing elderly population has created a marked growth in the need for long term care. Current estimates for expenditure predict a rapid escalation of healthcare costs as a proportion of the GDP of developed nations, yet at the same time a global economic crisis has necessitated dramatic cuts in health budgets. This unsustainable position has led to calls for an urgent transformation in healthcare systems.

This commentary explores the present day healthcare crisis and looks at the opportunities for chiropractors as pressure intensifies on politicians and leaders in healthcare to seek innovative solutions to a failing model. Amidst these opportunities, it questions whether the chiropractic profession is ready to accept the challenges that integration into mainstream healthcare will bring and identifies both pathways and potential obstacles to acceptance.

## Background

A need for transformation in healthcare systems throughout the globe has long been recognised [1-3]. Social reform, improvements in living conditions and the positive impact of public health initiatives have all conspired to enhance quantity and quality of life [4]. As the baby boomers of the post World War Two era move into their twilight years enjoying a range of activities that would have left their ancestors aghast [5], western societies have experienced a steady increase in the size of the ageing population as communities dance, jog, cycle and gyrate their way into their eighties and nineties [6].

But while we celebrate the achievements of medical science in prolonging and sustaining life, health care systems have been buckling under the pressure [7]. Advances in health technologies have brought about highly sophisticated systems of investigation, surgery and medical care [8,9]. In nations where health is delivered free at the point of service and where an informed public demands access to the most advanced available care, costs of health provision have rapidly escalated [10]. At the same time, fiscal deficits and global economic crises have resulted in budgets being dramatically reduced as governments struggle to balance the pressures on the public purse [11,12] whilst at the same time demanding added value. In nations which have seen the cost of healthcare as a proportion of the nation’s GDP rise steeply, in some cases by over seventy per cent [13], it is clear that within such an environment traditional models of service delivery are no longer fit for purpose.

## Discussion

### Global health crises

The impact of conflict between forced financial austerity and life-saving, though costly, technological advances in healthcare has been felt across the globe. The starkest example of the need for health systems transformation exists in the United States, where nearly 50 million residents live without insurance coverage [14]. Despite clear evidence supporting the need for change and the efforts of the current administration to put healthcare reform at the top of its agenda [15], political and establishment opposition has meant that progress has faltered to the point of stagnation [16]. Vested interests, notably of a powerful medical lobby and a dominant insurance industry have ensured that the public remain suspicious of any proposals for change. Calls for a synergy between government and large industry to reduce the burden [17] have still to produce meaningful results.

In the UK, the pressure under which the National Health Service has found itself has been compounded by national debt and a prolonged economic slump [18]. The coalition government which came to power in 2010 led to a wide ranging review of healthcare provision and a White Paper, *Equity and Excellence*: *Liberating the NHS*[19], set out the necessary steps for transforming the delivery of health care in the UK by setting out the need for a patient centred approach, the optimisation of outcomes, and the empowerment of clinicians to innovate in order to deliver the optimal, cost-effective services locally to their individual patient populations.

As a consequence, a new Health and Social Care Act [20] has been rapidly driven through the UK Parliament. The Act represents the biggest single reorganisation of the NHS since it was established in 1948. Despite a general acknowledgment that the current system was unsustainable, the measures contained in the Act have created much disquiet amongst mainstream health professionals [21] who under the reforms are set to be charged with independently controlling much of the £106bn healthcare budget [22].

By abolishing traditional budget-holding Primary Care Trusts and Strategic Health Authorities, the Act sets up one single Health Board (Public Health England) [23] to allocate resources and provide commissioning guidance. Under the new framework, family doctors are set to form local Clinical Commissioning Groups, with the power to prioritise budgetary spending to key identified areas of greatest health need and allocate resources on behalf of their patients as they see fit. The introduction of the Any Qualified Provider (AQP) scheme [24] allows those operating independently of the NHS framework to compete for contracts to deliver health services. Critics argue that the Act is complex, incoherent and not fit for purpose and have voiced fears that the inclusion of private individuals and organisations within service delivery will strike at the heart of an NHS that is cherished as a national public institution [25].

In Canada, while the public has expressed support for its nation’s health service [26], similar pressures to those seen in the UK have resulted in a growing clamour for transformation [27,28]. Here too, the growth of the aging population amidst a sharp increase in the cost and complexity of medicine and the burden of long term care for chronic conditions have left the system in crisis as administrations are faced with having to spread the healthcare budget ever more thinly [29].

Africa is faced with similar calls for health transformation, although here the drivers for change are frequently more associated with child mortality, HIV/AIDs and high levels of poverty [30]. Even a country such as South Africa, seen as wealthy within the context of its neighbours, faces significant health disparities [31]. Fragmented health services and inaccessibility outside of the main centres mean that a significant proportion of the population receive little in the way of modern healthcare [32].

Despite the protests of those who see the transformation of healthcare systems as being fraught with negative issues, not changing cannot be an option. Not only would maintaining the status quo provoke an economic catastrophe, human resource implications of continued growth and demand for services make the current system unsustainable. It is therefore imperative that innovative ways of satisfying need must be found and, if the prophecies of impending collapse are to be believed, transformation must occur swiftly.

### Opportunities for the chiropractic profession

For the chiropractic profession, the crisis provides opportunities hitherto unseen in its 117-year history. With an identified gap (some might say chasm) in the marketplace for cost-effective spine care, it is critical that these opportunities are understood.

The evolution of the profession to one that in many jurisdictions is subject to statutory regulation has meant that it should no longer be characterised as complementary or alternative medicine, but as a mainstream provider of neuromusculoskeletal services. Transformation gifts the profession opportunities to deliver solutions to critical issues of inadequate provision in the field of spinal health.

These opportunities present themselves in a range of fields. In the arena of public health, the provision of spinal health promotion can become the preserve of the chiropractic profession. Educating communities in posture, manual handling and basic self-help measures has the potential to position chiropractic as the experts in spinal health. In occupational medicine, where neuromusculoskeletal disorders are endemic, working with industry to reduce sickness absence and implement prompt return to work strategies has the potential for enormous savings to be made. In general medicine, working in multidisciplinary teams as spine care experts will assist in the provision of prompt evidence-based care and a reduced likelihood of chronic back pain through delayed access. Along with a focus on promoting health and wellness, mobility and independence within the older health sector can be supported by chiropractor-led teams.

As members of an established profession, chiropractors must rapidly wake up to the realisation that there is a void in the field of non-surgical spinal healthcare which chiropractic can fill. There must not, however, be any delay in ensuring that we are suitably prepared and able to respond in a way that makes chiropractic an attractive option for those charged with resolving the crisis in service provision. So far as health system transformation is concerned, the train is leaving the station and, as a profession, we must quickly decide whether we want to be on it. Not only that, we must also consider which class of ticket we wish to purchase and where in the train we would like to sit.

It would, of course, be inconceivable to think that chiropractic as a profession would not want to involve itself in the process of healthcare transformation. Certainly, there will be those who may seek to characterise such involvement as a malevolent shift toward a medicalisation of the profession and a loss of autonomy. These are frequently the same voices that caution against any dialogue with colleagues in other healthcare disciplines and who prefer the romanticism of a bygone era where accountability and clinical governance had not entered the chiropractic lexicon.

To grasp the opportunities at hand, chiropractors of all philosophical persuasions must recognise the critical importance of integration and multidisciplinary teamwork. There can be no place for isolationism in modern chiropractic. We can embrace what is happening in healthcare, see opportunities and become part of delivering a patient-centred model, or we can contract, shrivel up and accept that we were only ever destined to be jesters in the court of the musculoskeletal marketplace.

### Strategies for inclusion

Having discussed the opportunities for chiropractic within the transformation of healthcare systems it is necessary to explore what needs to be done to ensure that the profession is a key stakeholder.

Leaders in the profession have a responsibility and an obligation to ensure that chiropractic is well represented whenever there is talk of transformation in healthcare systems. Such representation must take place at all levels and there is a need to ensure that politicians recognise the benefits to society, the medical establishment realises that chiropractic can fill a void that no one else seems inclined to occupy, and chiropractors themselves share the vision that by uniting under a banner of universal access to patient-centred, evidence-based care, they can become the default experts in their field.

Chiropractic has the means to play a key role in healthcare transformation, but the profession does have to examine itself and overcome obstacles that have historically left it vulnerable to challenge and condemnation.

Perhaps first and foremost, there is a clear need to promote a consistent message. It is a matter of great regret that chiropractic’s identity is often blurred and the good work done by a hard working majority can very quickly be undone by a damaging minority of evangelists who preach a message of high volume, practitioner-centred practice building. In modern healthcare systems, there can be no place for treadmill chiropractic and organisations who seek to turn back pain into a profiteering industry cannot be allowed to flourish. While projects to define chiropractic’s identity have been previously undertaken, it may be timely to revisit this controversial area and decide whether in today’s environment this identity reflects the needs of a modern profession.

A strong case can be made for chiropractic, but words must be supported by actions. We must robustly condemn unethical practice, and no longer tolerate conduct which falls below acceptable standards of patient-centred care. If we are to play our part in the transformation of healthcare systems, our responsibility lies in raising the bar and ensuring that chiropractic is a profession deserving of serious consideration as a provider of mainstream healthcare.

Thanks to the researchers in our profession and to those who promote research, we can be confident in providing to those who are scrutinising our profession evidence upon which our claims of effectiveness are based. Evidence is of course the known truth at any one point in time, but evolutionary change in the profession must take place in order to survive and that change must in part be based on scientific research evidence.

Those committed to the development of the chiropractic profession have recognised the value of investment in research and there is an increasing number who have committed their careers to chiropractic research and who dedicate their working lives to ensuring that the profession is both academically recognised and respected. Through university-based affiliations, particularly in Europe and North America, the credibility of the entire profession is being steadily enhanced.

Modern, credible chiropractic researchers have entered a world which deals in finding out the truth and not being afraid of admitting that what chiropractors have done for many years may not be as good as they had previously thought. The publishing of negative as well as positive outcomes marks a maturing of the profession and better reflects the reality of healthcare.

For decades, the chiropractic profession has been blighted by myopic and defensive responses to research that challenged traditional opinions and approaches to care. That as a profession we can now be seen to be reflecting on what works and what does not and then evolving accordingly marks a maturing from an era of teenage angst. Not before time, it elevates the profession above those individuals who have traditionally misrepresented data and placed a positive spin on research in order to support an outdated and one-dimensional model of assessment and care.

### Modern chiropractic: a package of evidence-based care

For chiropractic to gain credibility, we must also accept that we cannot simply be one-trick ponies. Claims by some chiropractors that we should not be diagnosticians but merely the correctors of vertebral subluxation perpetuate the myth that consulting a chiropractor will invariably involve lengthy programmes of spinal manipulation. If we are to play a part in transformational health care we must promote the fact that chiropractors are primary healthcare practitioners who utilise a package of care grounded in evidence and in the interests of their patients.

We must move away from the popular perception that as soon as you step foot inside a chiropractor’s clinic you will be fast tracked into an extensive programme of spinal adjustments with or without objective evidence of improvement [33]. Fortunately, modern chiropractors are demonstrating by evidence-informed approaches and a broad range of clinical techniques that there is far more to chiropractic than spinal manipulation. The incorporation of other manual methods, exercise prescription and the promotion of active care through education all point to a patient centred care approach. The development of spinal care pathways [34,35] is increasingly seen as the future of effective management and chiropractors are ideally placed to participate in what has been historically referred to as ‘the Back Pain Revolution’ [36].

### Collaboration in multidisciplinary teams

In our transformational world, we must also see the potential for collaborative care. Effective healthcare systems, whether in Canada, the United Kingdom, Australia or elsewhere, rely on the sharing of best practice. This necessitates a mindset for collaboration and teamwork that places patients at the heart of care delivery and provides no place for interprofessional rivalry or petty squabbling. This does not mean there cannot be constructive debate, nor is there an obligation for health professionals always to agree. What is expected, however, is an environment of mutual respect where all parties can contribute freely and the best interests of the patient are placed at the forefront.

There are already examples of chiropractors involving themselves and setting up collaborative spine care teams. In hospital-based programmes, private clinics, and community initiatives, chiropractors are working within multidisciplinary groups. For the first time, chiropractors worked as part of a multi-faceted team within the host medical services Polyclinic at the London 2012 Olympic Games. This is the result of forward-thinking chiropractors who have professionally promoted the profession and who have defended the rights of chiropractors to be part of the greatest sporting event on the planet. In doing so, they have broken down barriers, corrected misapprehensions and have overcome pockets of resistance.

### Quality assurance in chiropractic healthcare

The acquisition of trust in any profession is underpinned by the quality of its education. The framework for quality assurance worldwide is provided by the Council on Chiropractic Education International (CCEI) [37]. Despite the existence of quality assurance agencies and regulatory authorities, there remains significant variation in chiropractic education across the globe, with some programmes emphasising a strongly evidence-based biomedical model of education whilst others prefer to promote a more traditional philosophically-centred approach.

While this disparity creates a rich diversity of practice within the profession, it nevertheless presents a confused message to those viewing the profession from the outside [38]. This variation may present an obstacle to the widespread acceptance of chiropractic into mainstream healthcare. While the evidence-based framework of one chiropractic degree programme may be wholly familiar to other health professions, the continued emphasis on theoretical subluxation-based models of care within other programmes leads to accusations that the evidence base for chiropractic care struggles to stand up to scrutiny [39]. A schism persists and there are growing calls to define where the future of the profession may lie [40].

We must therefore consider whether in our quest to achieve cultural authority in chiropractic our primary aim should be to ensure that there is a consistency in the delivery of education. At present, we have a somewhat anomalous situation where the portability of graduates between continents is hampered by wide variations in undergraduate education. In Europe, at the same time that Switzerland has a six year chiropractic programme leading to graduates being formally recognised as medical professionals [41], other programmes are being delivered that are far shorter and do not require full time attendance [42]. Of course we must acknowledge that modern education is outcomes- rather than hours-focused [43], but nevertheless, the gap between one programme and another and the entry requirements demanded of each suggests that not all chiropractors are the same. Styles of learning delivery can be problem-based and tutorial driven or a more traditional didactic approach [44], but if we are to compete and seize the opportunities that transformation may bring, decision-makers must be assured that minimum standards of education are being consistently delivered.

### A commitment to lifelong learning

In assuring both the public and other key stakeholders, we must also confirm a commitment to lifelong learning by making it a universal mandatory requirement [45]. Patients deserve and demand assurance that their health care providers remain up to date with developments in their field of expertise [46]. ‘Strongly encouraging’ continuing professional development in chiropractic is not enough and to stake our place as care-givers in the changing world of neuromusculoskeletal medicine we must show leadership by ensuring that chiropractors are mandated to maintain their skills and knowledge.

Primarily driven by moves to ensure that medical doctors remain up to date, the UK government is committed to developing systems of revalidation across all of the regulated health professions. What form revalidation may take for chiropractors is yet to be seen, but the objective is to ensure consistency and affirm good practice. Early consultations were marred by accusations of misleading and inaccurate extrapolations of risk data, but in promoting a patient-centred approach, the chiropractic profession should welcome a proportionate system of revalidation.

A standardised education, coupled with an obligation to undertake continuing professional development once in practice, must be considered two essential pillars in chiropractic’s quest for cultural authority. As a profession, we will be judged on the commitment to, and realisation of, high standards, continuous improvement and professional excellence.

### Chiropractors as non-surgical healthcare specialists

In transforming healthcare systems, it becomes governments to develop existing collaborative models and form new ones [47]. In doing so, they must commit to prioritising areas where the human and economic burden of disease is greatest, including obesity [48], diabetes [49], elderly long term care and spine care [50]. With respect to the latter, the failure of existing models of care to address the problem is of such magnitude that improving the provision of back and neck pain services must be a critical factor in any transformational health system design.

Across western societies, disproportionate costs are incurred by the small percentage of spine patients who develop chronic conditions [51]. It is estimated that it runs into billions of dollars, the true cost is difficult to define, as the financial burden of disease extends beyond the costs of providing health care [52]. The cost to industry, and to society through long term sickness benefits, and the costs associated with providing long term formal or informal care significantly magnify the true figures [53]. Ensuring prompt access must therefore be one of the key aims of any transformational strategy to prevent acute spinal conditions becoming unnecessarily chronic [54].

### Chiropractic’s duty to act

For chiropractic to demonstrate that it is ready to seize the opportunities that transformational healthcare systems may bring it needs to show that it has the capacity, the consistency and the capability to satisfy the needs of a nation and the requirements of collaborative partners.

Firstly, in terms of capacity, the chiropractic profession must ensure that it has sufficient numbers across a wide geographical distribution and population demographic to ensure prompt delivery of services. Human resourcing has been part of the problem in the existing model of care [55,56], so as a profession, chiropractic must ensure that it can deliver adequate numbers to satisfy demand. This may of itself require innovative models, such as that advocated by the World Health Organisation [57] and adopted by the chiropractic profession in Brazil [58].

Secondly, the chiropractic profession must deliver its services consistently by using internationally agreed models of care. Evidence-based practice, which employs best scientific information, clinical expertise and patient values and circumstances, must be embraced by chiropractors. There can be no place in such a model for extravagant programmes of care that encourage patient dependency and routinely overstate gravity. The profession must show its integrity in clearly defining those conditions for which it is most effective, and attention should be directed towards sub grouping [59,60]. By clearly setting out the patient groups within which chiropractic can be most effective, protocols for onward referral must be identified, so that referring physicians may be assured that when treatment is not working, patients may be either referred to another discipline or back to primary care. Classification systems have already been developed to enable effective targeting of subgroups and the outcomes have been encouraging [61].

Thirdly, we must show that we are capable of justifying our place in a new health system. Capability must be demonstrated by ensuring that as a profession we are equipped to effectively meet the expectations of patients, health service managers, politicians and other key stakeholders. This will require the profession to demonstrate that not only is it capable of delivering evidence based care in a timely manner; it must do so in such a way that maximises value for money [62].

Capacity, consistency and capability must all be underpinned by trust. Relationships between chiropractic and mainstream medicine have regularly been blighted by mutual suspicion and mistrust. For chiropractic to play a part in health system transformation, historical prejudices should now be set aside and a commitment must be made to work collaboratively in a spirit of openness and transparency.

There is already evidence of collaborative models that demonstrate that chiropractic is well placed to work within an integrated setting [63-65]. Hospital outpatient integration and other models of integrative healthcare should be championed. In the UK, a collaborative pilot study using chiropractors, osteopaths and physiotherapists resulted in a 28% reduction in referrals to the orthopaedic outpatient department and a satisfaction rate exceeding 90% amongst GPs and patients [66].

The size of the profession means that we should share these examples of best practice on a global level. The World Federation of Chiropractic [67] may be well placed to facilitate this process and ensure that collaborative models exist internally as well as externally. Similarly, the production of consensus statements and clinical practice guidelines demonstrate a commitment to quality patient care and a profession supportive of research-driven, evidence-based practice [68-70].

But it is one thing to put a system in place and another to make it work. The key to success will be the adoption of a chiropractic model of non-surgical spine care by the gatekeepers - the general medical practitioners. In the presence of credible alternatives, what is it that will convince MDs to take a leap of faith and refer a patient for chiropractic care?

Much as we would like to convince ourselves otherwise, the bottom line *is* the bottom line. It could be argued that we do not have to be any better than the competition; we just have to be cheaper. However, with pressures being applied at both ends and an existing model that has been woefully inadequate, there is clearly a need for chiropractic to be both better and cheaper if it is to be seen as an attractive option. And it does not end there. We need to be better, and cheaper, and have shorter waiting times, and be accountable at a much higher level than some of our counterparts. We have to prove ourselves worthy of any referral that comes our way and that means being at the top of our game as we contend with the last-chance saloon cases that may initially comprise much of our practice as we are tested by those wary of a profession seeking to stake its claim in an already-congested marketplace.

Our challenge lies with being equipped to deliver sustainable, high levels of service [71]. While established mainstream healthcare services have embraced the concept of clinical governance, chiropractors, as an autonomous group, have not universally embraced formal systems of clinical audit, outcome measurement, care pathways and protocols that help ensure sustainability. This is by no means an insurmountable hurdle yet it will require a shift in culture to one more in line with the system into which we aspire to be accepted [72].

## Conclusion

The transformation of healthcare systems provides many opportunities for chiropractic. There is now a good evidence base for the utility of the services that chiropractors provide and, coupled with the suboptimal management of non-surgical spinal disorders, chiropractors should be seriously considered as potential partners in the management of neuromusculoskeletal health.

Transformation in healthcare demands courage and leadership. It will mean plotting new courses and navigating difficult and treacherous waters. It will be brave leadership that decides that chiropractic constitutes part of the answer to the back pain crisis and it therefore befalls us to ensure that the decision is made easier by promoting a realistic, achievable model of care [73].

Just as chiropractic’s leaders have a duty to promote, develop and seize the opportunities that are available, so should we encourage and develop the concept of individual leadership in chiropractic. Chiropractic in western society may shortly be presented with unique opportunities which may not come again in our professional lifetimes. Recognising the opportunity is important. Seizing the opportunity is important. But it is sustaining the opportunity and delivering on expectations that will define who and what we become as chiropractors within the healthcare community.

## Competing interests

The author is the current President of the British Chiropractic Association.
